# A High Proportion of *Encephalitozoon cuniculi* Antibody-Positive Domestic Rabbits Identified in Northern Portugal: Implications for Zoonotic Risk

**DOI:** 10.3390/microorganisms14071510

**Published:** 2026-07-10

**Authors:** Filipa Rocha, Mark S. Gibson, Sofia Anastácio, Hugo Vilhena, Gonçalo Frouco, Sérgio Ramalho de Sousa, Joana Ferrolho

**Affiliations:** 1 Department of Veterinary Sciences, Vasco da Gama University School (EUVG), Avenida José R. Sousa Fernandes 197 Lordemão, 3020-210 Coimbra, Portugal; fiafilipar29@gmail.com; 2 Vasco da Gama Research Centre (CIVG), Department of Veterinary Sciences, Vasco da Gama University School (EUVG), Avenida José R. Sousa Fernandes 197 Lordemão, 3020-210 Coimbra, Portugal; mark.gibson@euvg.pt (M.S.G.); sofia.anastacio@euvg.pt (S.A.); hcrvilhena@hotmail.com (H.V.); goncalo.frouco@euvg.pt (G.F.); sergio.sousa@euvg.pt (S.R.d.S.); 3 Center of Neurosciences and Cell Biology, Health Science Campus, 3000-548 Coimbra, Portugal; 4 Department of Veterinary Clinics, School of Medicine and Biomedical Sciences, ICBAS-UP, University of Porto, 4050-313 Porto, Portugal; 5 Animal and Veterinary Research Centre (CECAV), University of Trás-os-Montes and Alto Douro (UTAD), Quinta de Prados, 5000-801 Vila Real, Portugal; 6 Associate Laboratory for Animal and Veterinary Science—AL4AnimalS, 1300-477 Lisboa, Portugal; 7 Center for Studies in Animal Science (CECA), Fernando Pessoa University, Praça 9 de Abril 349, 4249-004 Porto, Portugal

**Keywords:** *Encephalitozoon cuniculi*, microsporidia, ELISA, domestic rabbits, zoonosis, Portugal

## Abstract

*Encephalitozoon cuniculi* is a globally distributed microsporidian of veterinary and public health relevance, particularly due to its zoonotic potential and high prevalence in domestic rabbits (*Oryctolagus cuniculus*). This study aimed to estimate the presence of anti-*E. cuniculi* antibodies in pet rabbits from the Porto district, Portugal, using an indirect enzyme-linked immunosorbent assay (ELISA). Blood samples were collected from 54 domestic rabbits during routine veterinary appointments and hospitalisation between September 2024 and January 2025. Plasma samples were analysed for anti-*E. cuniculi* antibodies. Thirty-four rabbits (63%) were seropositive, indicating substantial exposure within the sampled population. This prevalence is consistent with reports from other European countries and may indicate *E. cuniculi* is endemic among pet rabbits in Portugal. Given the zoonotic nature of this pathogen, these findings reinforce the need for surveillance strategies integrating animal and public health perspectives.

## 1. Introduction

Rabbits (*Oryctolagus cuniculus*) are a highly versatile species, contributing significantly to the economies of many countries. Europe ranks as the second-largest producer of rabbit meat globally, reflecting the species’ economic and nutritional importance [1].

In addition to their agricultural role, rabbits are extensively used in biomedical research [2].

Rabbits are also among the first animals many people acquire as pets [3]. Improved husbandry and healthcare have extended their lifespans, increasing the frequency with which rabbits attend veterinary clinics. This closer interaction between humans and rabbits raises important public health considerations, particularly regarding zoonotic pathogens such as *Encephalitozoon cuniculi*, which poses a risk to immunocompromised individuals, including children, the elderly, and those undergoing medical treatments such as chemotherapy or organ transplantation [4]. Veterinary professionals and laboratory workers are also potentially exposed, highlighting the need for early and accurate diagnosis to ensure effective treatment, prevent complications, and reduce transmission.

Parasitic diseases represent a critical concern for animal welfare and productivity. Infected rabbits are more susceptible to secondary infections due to immunosuppression, and severe or untreated parasitic infections may result in mortality. Even subclinical parasitism can adversely affect reproductive performance and growth in production animals [5]. *Encephalitozoon cuniculi* is an obligate intracellular microsporidian, historically classified as a parasite (previously *Nosema cuniculi*) and now considered a fungus-related pathogen. First described in 1922 in young rabbits exhibiting granulomatous encephalitis and motor paralysis, *E. cuniculi* is a globally distributed zoonotic pathogen affecting both animal and human populations [4,6].

Microsporidia, including the genus *Encephalitozoon*, comprise over 1200 species, 17 of which infect humans. The parasite gained prominence during the 1980s due to its association with AIDS, where immunosuppression facilitated systemic spore dissemination in humans. Other vulnerable groups include cancer patients, organ transplant recipients, diabetics, children, the elderly, and occupationally exposed individuals, such as those working with animals or in slaughterhouses. Clinical manifestations in humans are diverse, ranging from fever, diarrhoea, and respiratory, ocular, or nasal symptoms to encephalitis, nephritis, hepatitis, keratoconjunctivitis, and cognitive impairments. In immunocompetent individuals, infection is often subclinical [7]. The parasite’s low host specificity, high environmental resistance, and capacity to cause severe disease underscores the need for research and management within a One Health framework.

*Encephalitozoon cuniculi* exhibits two primary routes of transmission in rabbits: vertical transmission, confirmed through the detection of spores in placentas and foetuses leading to intraocular infections, and horizontal transmission, primarily via ingestion or inhalation of spores excreted in urine or faeces [8,9]. Spores may remain viable for several weeks in the environment, and infection begins when the spore’s polar filament injects sporoplasm into host cells, initiating merogony and subsequent sporogony, culminating in infective spores that are shed and capable of systemic dissemination [4]. *E. cuniculi* is classified into four genotypes (I–IV), with geographic and host-specific variations influencing prevalence patterns. While genotype I predominates globally in the rabbit population, genotype II has been reported in Slovakia and China and genotype III only in China in domestic rabbits [10]. Documented cases of genotype IV infecting rabbits are absent in the literature.

Clinical signs in rabbits are often neurological, ocular (phacoclastic uveitis), or renal (chronic interstitial nephritis), with vestibular dysfunction being the most easily recognised by owners. Disease manifestation can be triggered by stress and is more likely in immunocompromised or young and elderly rabbits. Diagnosis relies predominantly on serology (ELISA, IFAT, CIA), while PCR may produce false negatives due to intermittent spore shedding. Treatment strategies focus on inhibiting spore proliferation (e.g., fenbendazole), anti-inflammatory therapy, supportive care, and physiotherapy, while preventive measures emphasise serological screening, quarantine, and environmental hygiene [7,9,11]. Despite a wealth of publications on this pathogen in rabbits (reviewed in [10]), epidemiological data from Portugal remains scarce. To date, *E. cuniculi* has only been documented in humans, but not in animals, in Portugal [12,13]. Only two previous studies, both master’s degree dissertations, have assessed the presence of anti-*E. cuniculi* antibodies in rabbits in Portugal. One used a carbon immunoassay, identifying 26 positive animals from the 55 tested (42.7%) in the Lisbon region [14]. The other study used antibody titration, reporting 87.5% of the animals were positive, and further determining 82.1% of these infections were chronic and 17.9% were active. Thirty-two rabbits, also from the Lisbon region, were evaluated in this study [15]. The present study aimed to estimate the exposure rate of *E. cuniculi* in domestic rabbits from the Porto district, contributing novel data relevant to pathogen surveillance and zoonotic risk assessment.

## 2. Materials and Methods

### 2.1. Ethics Statement

This study was approved by the Ethics Committee of the Vasco da Gama University School (CE-EUVG; reference no. 26/2024), Coimbra, Portugal.

### 2.2. Study Design and Sampling Approach

A cross-sectional survey was conducted from September 2024 to January 2025. Convenience blood sampling from rabbits was carried out during routine appointments and hospitalisation at Be Exotic—Veterinary Clinic, Matosinhos, Porto, Portugal (https://www.beexoticvet.pt/), without pre-established clinical criteria, and no additional patient data were recorded. Whole blood was collected into lithium heparin-coated tubes (Greiner Bio-One ACU TT^TM^ Heparin Blood Collection Tubes; Greiner Bio-One, Kremsmünster, Austria) as part of the diagnostic plan for each animal. To isolate plasma, the samples were centrifuged at 4500 rotations per minute (rpm) for 8 min at room temperature (VWR Micro Star 30R refrigerated centrifuge; VWR, Carnaxide, Portugal). Following centrifugation, plasma was used for biochemical analysis. The remaining amount of plasma was then transferred into individually labelled microcentrifuge tubes for this study and stored at −20 °C until required for analysis.

### 2.3. Sample Analysis

#### 2.3.1. Sample Preparation

All plasma samples were analysed at the Vasco da Gama Research Centre (CIVG), Vasco da Gama University School (EUVG), Coimbra, Portugal. Samples were transported frozen from the clinic in Porto to the CIVG in Coimbra to ensure sample integrity prior to laboratory processing.

#### 2.3.2. Enzyme-Linked Immunosorbent Assay (ELISA)

The presence of anti-*E. cuniculi* antibodies in rabbit plasma was assessed using the *Encephalitozoon cuniculi* Antibody ELISA Test Kit (Medicago Group, Uppsala, Sweden; product code: 18-9001-1), which employs indirect methodology. In this assay, *E. cuniculi*-specific primary antibodies present in the plasma bound to antigen-coated wells in the microwell plate during an initial incubation. After washing to remove unbound material, a Horseradish Peroxidase-labelled secondary antibody was added, forming a detectable antigen–antibody complex. The remainder of the assay was completed following the manufacturers protocol, with absorbance measured at 450 nm using a Multiskan FC Microplate Photometer (Thermo Scientific^TM^, Thermo Fisher Scientific, Waltham, MA, USA).

Samples were analysed in quadruplicate: two wells coated with *E. cuniculi* antigen and two wells with a non-specific control antigen. Positive and negative controls were included in each assay to validate the unknown samples. The serological status of each sample was determined by dividing the mean OD in *E. cuniculi* antigen-coated wells by the mean OD in control antigen-coated wells. Samples were considered positive for anti-*E. cuniculi* antibodies when the result exceeded 2, as indicated in the ELISA Test Kit protocol.

### 2.4. Statistical Analysis

Presence of anti-*E. cuniculi* antibodies in domestic rabbits was calculated as the proportion of positive samples among the total tested, expressed as a percentage. The 95% confidence interval (CI) was calculated (normal approximation method, *z* = 1.96). The Standard Error (SE) and the Margin of Error (MOE) were determined using standard formulae.

## 3. Results

Thirty-four of the fifty-four plasma samples (63%; 95% CI [50.1, 75.8]) tested positive for anti-*E. cuniculi* antibodies, while twenty samples (37%) were negative. All positive and negative controls met the validity criteria specified by the manufacturer. No inconclusive results were observed. To assess assay precision, samples were tested in duplicate (per antigen) on the same ELISA plate. Intra-assay variability was calculated as the coefficient of variation (CV) of these duplicate measurements. The mean intra-assay CVs on the three ELISA plates used were 3.57% (0–20), 9.16% (1.11–25.22), 1.96% (0.07–6.11), indicating good repeatability. Inter-assay CVs were calculated from the ratios between the specific and control antigen-coated plates for the negative and positive control samples across the three ELISA plates used. Each type of antigen and each control sample was tested in duplicate. Inter-assay CVs were 27.47% and 12.62% for the negative and positive control samples, respectively.

## 4. Discussion

This study provides evidence of the presence of *E. cuniculi* among domestic rabbits in the Porto district, Portugal, with an antibody prevalence of 63% detected via ELISA. This previously unknown prevalence was situated at the higher end of reported ranges in Europe and globally [10], though clinical metadata on the animals we tested, and methodological alignment would be required before we could viably compare our results with other studies. A direct comparison with published seroprevalence estimates is challenging because reported values vary substantially between countries and regions. Geographical variation may reflect differences in climate, the environmental persistence of spores, rabbit density per region, management practices, sanitation, and biosecurity. All these factors can influence horizontal transmission and environmental exposure [16,17]. Differences in the temporal period during which studies were conducted may also contribute to variation, as management practices and awareness of *E. cuniculi* have altered over time [17]. Population characteristics further complicate comparisons. The literature includes studies involving pet rabbits, breeding colonies, commercial meat rabbits, laboratory rabbits, rescue populations, or mixed cohorts, each representing distinct demographic and management conditions [4,18]. Variations in age, housing systems, stocking density, animal movement, and contact with other rabbits may influence exposure risk and therefore reported seroprevalence. Differences in inclusion criteria, such as sampling only “clinically healthy” rabbits, or randomly assigned animals presented to veterinary practices such as in our study, may also affect prevalence estimates [17,19]. Methodological heterogeneity represents an additional source of variation. Studies have employed ELISA, indirect immunofluorescence antibody tests (IFAT), carbon immunoassays, modified agglutination tests, and PCR, each with different analytical sensitivity, specificity, and diagnostic targets. Even among ELISA-based studies, differences in antigen preparation and source, assay validation, positivity thresholds, and interpretation criteria may affect the data reported. Sampling conditions, including sample size, sampling strategy, geographical coverage, and whether samples were collected from single or multiple locations, also influence the accuracy and precision of prevalence estimates [20,21].

Notably, there are no peer-reviewed reports of anti-*E. cuniculi* antibodies in domestic rabbits in Portugal. Though, two previous studies, carried out as dissertations within master’s degrees, assessed the prevalence of *E. cuniculi* within the Lisbon region of Portugal [14,15]. Both studies used alternative methods of quantification, specifically a carbon immunoassay (CIA) [14] and antibody titration [15], making a direct comparison with our ELISA-based data impractical. They reported an anti-*E. cuniculi* antibody prevalence of 42.7% from 55 animals tested [14] and 87.5% from 32 animals, of which 82.1% were chronic and 17.9% were active [15]. These two studies show clear evidence of prior exposure to *E. cuniculi* in the Lisbon region, which in addition to our findings from Porto, over 300 km away, suggest widespread prevalence nationally. Understanding seroprevalence in domestic rabbits is crucial for informing early detection, improving clinical outcomes, and mitigating risks to both rabbit populations and vulnerable human groups.

Quantification by ELISA facilitated sensitive detection of anti-*E. cuniculi* antibodies; however, the kit used in this study is unable to differentiate between immunoglobulin classes (IgM vs. IgG) or quantify titres, which limits the capacity to distinguish recent from past exposure. Despite this limitation, antibody detection alone indicates historical evidence of prior exposure to the microsporidian parasite and reinforces concerns regarding vertical transmission and environmental contamination through excretions [4].

This study focused exclusively on pet rabbits, a population of increasing popularity [3], particularly among children in Portugal. The absence of mandatory regulations requiring vaccination, deworming, or health screening for *E. cuniculi* in these animals presents a dual concern: potential compromise of animal welfare and heightened risk of zoonotic transmission to vulnerable human populations, including immunocompromised individuals. Given the potential for both vertical transmission and environmental contamination via excretions, domestic rabbits may act as a silent reservoir for the parasite [7].

Several limitations must be acknowledged. The relatively small sample size (*n* = 54) and the recruitment of animals from a single veterinary clinic may introduce selection bias and limit how accurately the findings represent the national picture. Furthermore, the absence of detailed clinical and demographic information—including age, sex, health status, and medical history—constrained the analysis of host-related risk factors. Inclusion of such variables in future studies would allow a more nuanced understanding of exposure dynamics, particularly regarding subclinical cases.

From a public health perspective, these findings support the implementation of routine serological screening for *E. cuniculi*, ideally using an assay that could accurately distinguish between active infection (IgM) and past infection/exposure (IgG). A positive IgM result may carry a risk of zoonotic transmission with active spore shedding and may require drug treatment prior to the sale or adoption of rabbits. A positive IgG result would require further investigation and may not require additional measures. Complementary strategies should include pre-breeding testing, isolation of new or high-risk animals, and strict biosecurity measures. Education campaigns targeting breeders, veterinarians, pet shop staff, and owners are essential to raise awareness of exposure risks and reinforce preventive measures, in line with a One Health approach [7]. Our data underscores the pathogen’s presence and its potential public health implications in Portugal. Given the zoonotic nature of *E. cuniculi*, which can infect immunocompromised humans and a wide range of animal species, these findings highlight the need for enhanced surveillance and proactive management strategies in both veterinary and public health contexts [22,23].

Ongoing research is critical to fully characterise *E. cuniculi* infections. Priorities include longitudinal studies on disease progression, genotypic characterisation of local strains, validation and optimisation of diagnostic methods, investigation of zoonotic spillover to humans, assessment of treatment efficacy, and evaluation of environmental decontamination protocols [7,10].

## 5. Conclusions

The present study demonstrates a relatively high proportion of *Encephalitozoon cuniculi*-positive rabbits in the Porto district, Portugal. These findings underscore the importance of implementing routine serological screening and reinforcing preventive measures in pet rabbit populations. We did not collect data on the sampled animals, hindering epidemiological interpretation. This meant potential risk factors, disease associations, and sampling bias could not be evaluated. With regard to external validity, many more samples, harvested randomly, and from a broader geographical area, are essential. The population sampled in this study may not be representative of the national picture. Further large-scale studies integrating clinical, demographic, and molecular data are needed to characterise the epidemiology of *E. cuniculi* and to inform veterinary and public health strategies within Portugal.

## Data Availability

The original contributions presented in this study are included in the article. Further inquiries can be directed to the corresponding author.

## Abbreviations

The following abbreviations are used in this manuscript:

*E. cuniculi*	*Encephalitozoon cuniculi*
ELISA	Enzyme-Linked Immunosorbent Assay
OD	Optical Density
CIA	Carbon Immunoassay
IFAT	Indirect Fluorescence Antibody Test
PBS	Phosphate-Buffered Saline
PBS-T	Phosphate-Buffered Saline with 0.05% Tween 20
TMB	3,3′,5,5′-Tetramethylbenzidine
IRB	Institutional Review Board
